# The inferior vena cava (IVC) syndrome as the initial manifestation of newly diagnosed gastric adenocarcinoma: a case report

**DOI:** 10.1186/s13256-015-0696-3

**Published:** 2015-09-28

**Authors:** Shyam A. Patel

**Affiliations:** Department of Medicine, Stanford Hospital and Clinics, Stanford University School of Medicine, 300 Pasteur Drive, Stanford, CA 94305 USA

**Keywords:** Gastric adenocarcinoma, Hepatic metastasis, Inferior vena cava, Paraneoplastic syndrome, Secondary prevention, Superior vena cava

## Abstract

**Introduction:**

Vena cava compression is a relatively rare initial manifestation of underlying malignancy. The superior vena cava syndrome, which is characterized by facial plethora, jugular venous distension, and arm swelling, is a well-known entity associated with bronchogenic carcinoma. Less common is the compression of the inferior vena cava. To the best of my knowledge, this is the first reported case of newly diagnosed gastric adenocarcinoma presenting initially as the inferior vena cava syndrome. The unique aspect about this case is that it highlights a rare presentation before diagnosis of gastric adenocarcinoma.

**Case presentation:**

A 56-year-old Malaysian woman with a past medical history of iron deficiency anemia presented with lower extremity edema and progressive fatigue of 1 month’s duration. She had significant worsening of leg swelling after standing for short periods of time. She also reported epigastric discomfort, which led to an additional workup, including computed tomography of the abdomen and pelvis. This revealed a 3cm×2.9cm mass in the stomach, extensive hepatic metastasis, and severe inferior vena cava compression. The patient was examined further with esophagogastroduodenoscopy, and a biopsy showed gastric adenocarcinoma.

**Conclusions:**

This report describes a case of a patient with inferior vena cava syndrome as a unique presentation of previously undiagnosed stage IV gastric adenocarcinoma. Patients presenting with inferior vena cava syndrome should undergo prompt evaluation for underlying malignancies that have a predilection for hepatic metastasis. This case is important because earlier recognition of this syndrome can lead to earlier workup and thus detection of malignancy. Prompt initiation of treatment, including chemotherapy or vena cava stent placement, can result in improved patient outcome.

## Introduction

The clinical manifestations of malignancy vary tremendously and can include direct mass effect of tumor and paraneoplastic effects. A paraneoplastic syndrome is frequently the first manifestation of malignancy. Common paraneoplastic manifestations of gastric adenocarcinoma include acanthosis nigricans and Leser-Trélat sign [1, 2]. Other features that are mostly unique to gastric cancer include Virchow’s node, Krukenberg tumor, and Sister Mary Joseph nodule, though these are not common at the time of initial presentation [3, 4]. Direct mass effect is typically detected during clinical examination only after there is significant imposition upon other anatomic structures, such as nerves or vessels. For example, symptoms such as facial plethora and arm swelling as a result of the superior vena cava (SVC) syndrome in a patient with a history of smoking sometimes leads to the workup and eventual diagnosis of lung cancer. An underlying cancer is the cause of more than 90% of cases of the SVC syndrome. Other cancers associated with SVC syndrome include thymoma, mesothelioma, lymphoma, and germ cell tumors [5]. However, the clinical presentation of these malignancies does not include vena cava compression in most cases.

There are very few reports of inferior vena cava (IVC) compression, in contrast to the SVC syndrome, as a manifestation of underlying malignancy. Some tumors have been reported to have compressive effects on the IVC. A recent case report showed that IVC compression was caused by a mesenteric desmoid tumor in a patient who presented with acute pulmonary embolism [6]. Neuroblastoma in a neonate who had lower extremity edema was shown to cause IVC compression [7]. Two cases of paragangliomas have been reported to cause IVC compression posteriorly [8].

## Case presentation

A 56-year-old Malaysian woman with a past medical history of anemia presented with lower extremity swelling, early satiety, and abdominal discomfort of 1 month’s duration. She reported worsening of leg edema upon standing for short periods of time. She denied chest pain or shortness of breath. She had emigrated from Malaysia approximately 10 years before presentation. She had been taking oral iron supplementation and omeprazole. A colonoscopy had been performed 1 year before presentation to assess for gastrointestinal bleeding, and the results were normal. Her *Helicobacter pylori* breath test result was positive, and she was started on triple therapy with two antibiotics plus a proton pump inhibitor. An endoscopic examination was done, and the result was suggestive of a mass, though the diagnosis was not definitive. Her family history was not significant. Her social history was significant for frequent consumption of fish in Malaysia and was negative for alcohol, cigarette, or drug use.

Upon her admission to Stanford University Hospital, she reported a 10-lb weight gain. The weight gain was caused by edema, though she felt a sensation that she was becoming thinner. She reported that she was unable to stand for more than 15 minutes owing to accumulation of fluid in her legs. She reported that her mobility was thus limited. Her vital signs were stable, with a body temperature of 37°C, blood pressure of 134/78mmHg, pulse of 79 beats per minute, and respiratory rate of 18 breaths per minute. Her O_2_ saturation was 100%. Her physical examination revealed a grade 3/6 systolic ejection murmur heard best at the left lower sternal border. Her abdomen was non-tender to palpation, and her bowel sounds were normoactive. Examination of the extremities revealed 2+ non-pitting edema extending to her thighs bilaterally. She had no palpable peri-umbilical or supraclavicular nodes. She had no other palpable lymphadenopathy. Her skin examination was normal. Laboratory values showed hemoglobin of 8.5g/dl with mean corpuscular volume of 71.2fl. Her reticulocyte count was 4.36%. An iron panel revealed ferritin of 32ng/ml, transferrin of 403μg/dl, transferrin saturation of 3%, and iron level of 13μg/dl. Her platelet count was 519,000/μl, and her white blood cell count was normal. Her metabolic panel was significant for sodium of 134mmol/L. Her liver function tests were significant for aspartate aminotransferase of 122U/L, alkaline phosphatase of 478U/L, and albumin of 2.3g/dl. Her electrocardiogram showed T-wave inversions in leads V1–V4 and an S1Q3T3 pattern.

**Fig. 1:**
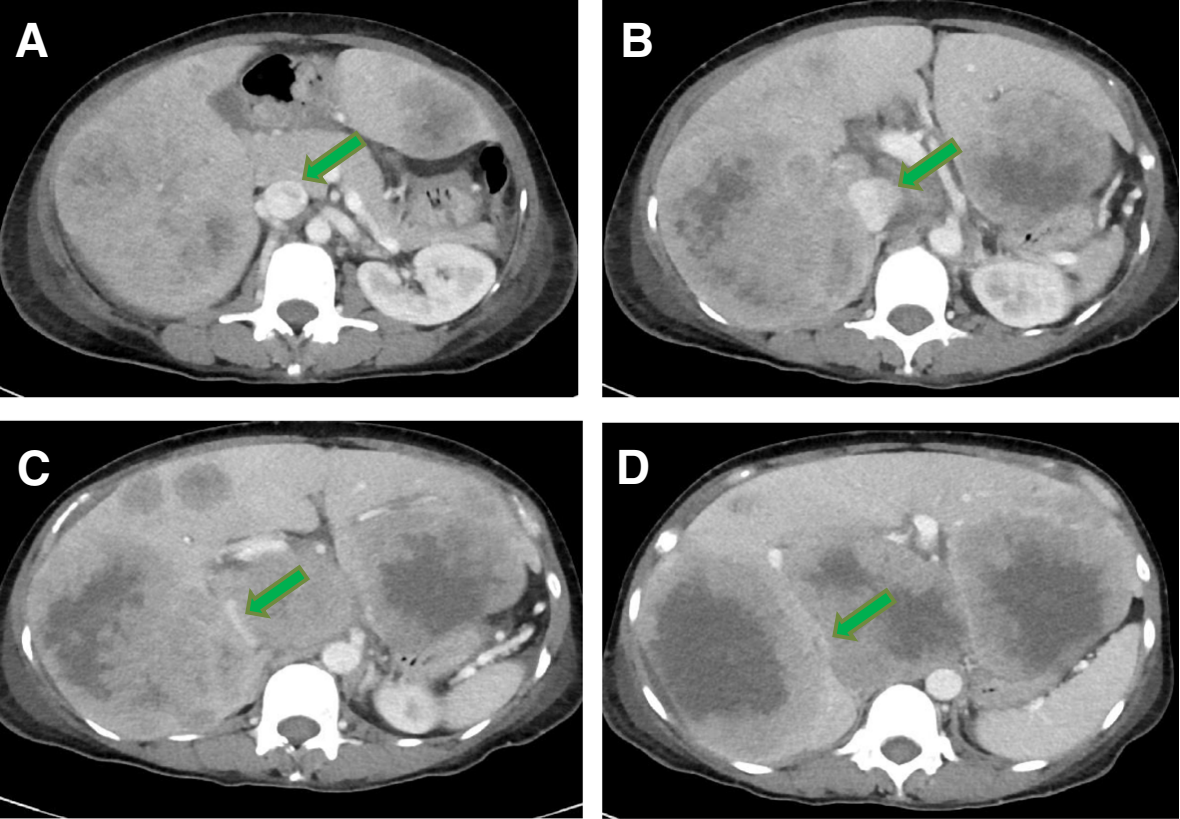
Compression of the inferior vena cava by foci of hepatic metastases. Computed tomographic scan of the abdomen and pelvis with intravenous contrast shows that the inferior vena cava (*green arrows*) becomes progressively more compressed by the tumor superiorly to inferiorly. **a** Patent inferior vena cava at the level of the pancreas. **d** Near-complete compression of the inferior vena cava at a level of the superior portion of the kidneys. **a**-**d** Superiorly to inferiorly

**Fig. 2:**
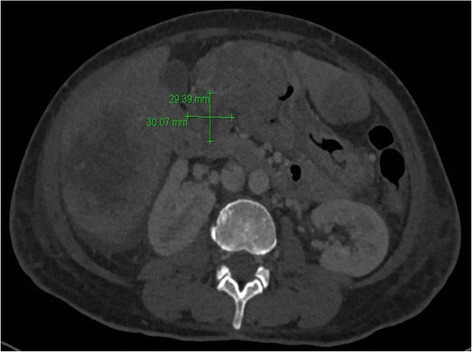
Adenocarcinoma involving the antrum of the stomach. Computed tomographic scan of the abdomen and pelvis with intravenous contrast shows an ill-defined 3cm×2.9cm necrotic mass. Scan also reveals omental implants and nodular extension of the gastric tumor posteriorly into the lesser sac

**Fig. 3:**
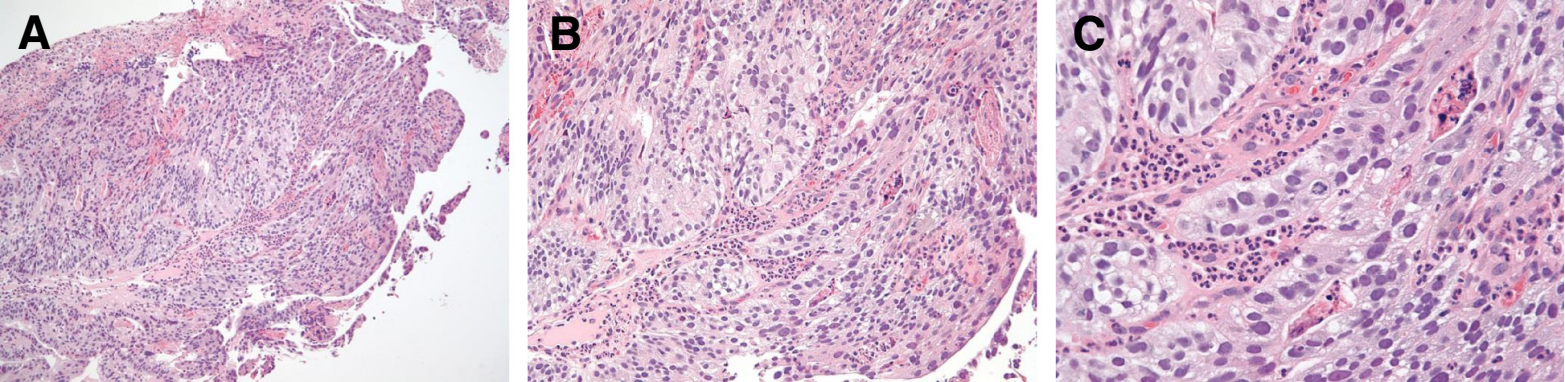
Histologic specimens from gastric antral mass biopsy. The biopsy revealed invasive, moderately differentiated adenocarcinoma of intestinal type, with formation of glands and nests. Shown are images at various magnifications: ×10 (**a**), ×20 (**b**), and ×40 (**c**)

The differential diagnosis included pulmonary embolism and myocardial injury or ischemia. Her troponin level was negative. An ultrasound of the lower extremities showed no deep vein thrombosis. Computed tomography (CT) of the abdomen and pelvis revealed severe intrahepatic narrowing of the IVC due to mass effect (Fig. 1) and an ill-defined 3cm×2.9cm mass involving the antrum of the stomach (Fig. 2). The CT scans were also notable for omental implants and nodular extension of the gastric tumor posteriorly into the lesser sac. She had a small amount of ascites. The gastric mass was biopsied during endoscopy (initially at an outside hospital). The histologic sections of the gastric antrum mass biopsy revealed an invasive, moderately differentiated adenocarcinoma of intestinal type, with formation of glands and nests (Fig. 3). The biopsy had a pancytokeratin-positive neoplasm. The patient was started on chemotherapy with capecitabine 1500mg by mouth twice daily and oxaliplatin 130mg/m^2^. The interventional radiology team evaluated the patient for possible IVC stent placement at a later time with the goal of alleviating venous obstruction. IVC stent placement was deferred while the patient was an inpatient because this procedure was typically reserved as palliative treatment for patients in whom chemotherapy has limited utility or for patients who have a poor response to chemotherapy.

**Fig. 4:**
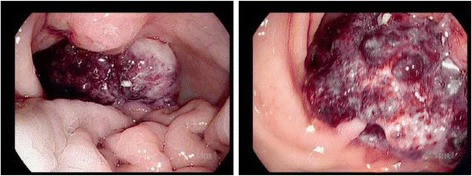
Endoscopic images of gastric tumor

She was followed in the outpatient oncology clinic and was scheduled for a total of six cycles of capecitabine with oxaliplatin. Her course was complicated by gastrointestinal bleeding from the gastric tumor after the fourth cycle of chemotherapy (Fig. 4). Esophagogastroduodenoscopy revealed a tumor that was unamenable to endoscopic therapy. She then recovered from gastrointestinal bleeding. Her HER2 test result was positive before the continuation of additional cycles of capecitabine and oxaliplatin, and she was thus started on trastuzumab 8mg/kg and paclitaxel 175mg/m^2^. She reported improvement in her appetite and energy level at her follow-up visits, though she continued to have abdominal bloating. At the time this writing, she was in cycle 5 of paclitaxel and trastuzumab.

## Discussion

The initial presentation of gastric adenocarcinoma typically involves abdominal symptoms and hematologic abnormalities, including anemia due to gastrointestinal blood loss. Paraneoplastic signs are sometimes the initial manifestations. One case report of a man with a new diagnosis of gastric adenocarcinoma revealed that the first sign was multiple seborrheic keratoses on the shoulders [2]. It is worthwhile to note and appreciate the less common presentations of internal malignancy, as these may comprise the first and only indication of early disease. Early diagnosis, especially in cases of malignancy, can divert poor clinical outcomes. Vena cava compression as a result of malignancy can be a challenge to diagnose and manage. The SVC syndrome is a well-documented feature of lung cancer, but there exist few case reports of patients presenting with symptoms related to mass effect on the IVC. IVC compression is interestingly not limited to malignancies; other causes include thrombus and intrinsic caval disease, which must be considered when performing a workup for cancer [9].

The diagnosis of the IVC syndrome is based largely on clinical features, including lower extremity edema, in the setting of radiographic findings demonstrating vena cava compression. Tachycardia can result from decreased preload due to venous pooling in the lower extremities. Imaging modalities in the past have included radionuclide venography. Current methods include CT of the abdomen, which can reveal intracaval thrombi, and magnetic resonance imaging with angiography [9, 10].

The treatment of vena cava compression syndromes commonly involves stenting or radiation. Expandable metallic stents have been used to treat IVC compression caused by hepatic tumors [11]. Tumors that compress the SVC, such as lung cancer, are generally radiosensitive [12]. In contrast, treatment of the IVC syndrome has not been clearly described, but presumably it involves treating the underlying cause of compression. This may include a combination of surgery, chemotherapy, and radiation. Interventional approaches include placement of an intravascular stent or surgical bypass grafting [5]. The patient was noted to have significant improvement in her lower extremity edema after initiation of capecitabine and oxaliplatin, likely resulting in reduction in the size of the metastatic lesions.

To the best of my knowledge, this is the first reported case of a patient presenting with lower extremity edema and the IVC syndrome as a manifestation of underlying stage IV gastric adenocarcinoma. There are some well-known paraneoplastic manifestations of gastric adenocarcinomas, but IVC compression has not been previously described. This case report emphasizes that workup of underlying malignancy, especially primary cancers with predilection for liver metastasis, should be done in patients with signs and symptoms of IVC compression.

## Conclusions

This is a significant case because prompt detection of malignancy can lead to improved outcomes. This case highlights the important components of secondary prevention in gastrointestinal oncology—namely, prevention of complications or exacerbation of disease once the disease is diagnosed. In this case, an improvement in outcomes was achieved by having a low threshold for initiating a workup for malignancy in a patient who presented with the IVC syndrome.

## Consent

Written informed consent was obtained from the patient for publication of this case report and any accompanying images. A copy of the written consent is available for review by the Editor-in-Chief of this journal.

## Abbreviations

CT: computed tomography
IVC: inferior vena cava
SVC: superior vena cava
